# A multiple-trainee, multiple-level, multiple-competency (multi-TLC) simulation-based approach to training obstetrical emergencies

**DOI:** 10.1007/s40037-019-00534-7

**Published:** 2019-10-07

**Authors:** Valerie Mueller, Susan Ellis, Beth Murray-Davis, Ranil Sonnadara, Lawrence E. M. Grierson

**Affiliations:** 1 Department of Obstetrics and Gynecology, McMaster University, Hamilton, ON Canada; 2 Midwifery Education Program, McMaster University, Hamilton, ON Canada; 3 Department of Surgery, McMaster University, Hamilton, ON Canada; 4 Department of Family Medicine, McMaster University, Hamilton, ON Canada; 5 McMaster Faculty of Health Sciences Program for Education Research, Innovation, and Theory, McMaster University, Hamilton, ON Canada

**Keywords:** Competency-based medical education, Simulation, Assessment, Obstetrics

## Abstract

Competency-based education requires that programs increase the breadth of direct observation and assessment to improve resident training. To achieve these goals, the authors developed and executed a multiple-trainee, multiple-level, multiple-competency (Multi-TLC) obstetrical emergencies simulation curriculum. Depending upon their training level (PGY1–PGY5), obstetrics and gynaecology residents participated in various roles (i.e., first responder, second responder, confederates, and evaluators) within four simulation scenarios designed to provide opportunities for education, direct observation, and assessment across a number of competencies (i.e., medical expert, communicator, collaborator, leader, advocate, and scholar). The curriculum was carried out over 8 h spread evenly across 2 days (i.e., 4 h/day) and involved periods of pre-briefing, live simulation, and debriefing. An evaluation of the Multi-TLC was operationalised via a context-input-process-product model. This report presents the outcomes of that evaluation derived from quasi-experimental comparisons of the new and previous curricula across four priorities for simulation-based education identified by the Department of Obstetrics and Gynecology at McMaster University (Hamilton, ON, Canada): increasing learning opportunities, maintaining or improving resident learning, maintaining or reducing program costs, and improving resident satisfaction. The evaluation revealed that the Multi-TLC curriculum permitted a greater breadth of direct observation and assessment across competencies, maintained the previous learning objectives while also addressing additional ones, and was done so in a way that reduced the overall financial and human resource costs associated with the department’s obstetrical emergency simulation curriculum. A Multi-TLC organisation of simulation curricula can facilitate efficient application of competency-based education principles.

**Electronic supplementary material:**

The online version of this article (10.1007/s40037-019-00534-7) contains supplementary material, which is available to authorized users.

## Introduction

Competency-based education demands that programs underpin entrustment decisions with sufficient observations to justify progression along each stage of the continuum [1]. These observations are largely conducted while residents are directly engaged with patients. Although, for competence associated with high-stakes procedures and rare emergencies, simulation provides valuable opportunities to observe and assess residents. However, simulation training is constrained by limited finances and faculty time. As more simulations are needed to observe more residents across more domains of competent practice, these costs will balloon.

In order to boost efficiency without compromising effectiveness, the Department of Obstetrics and Gynecology (OBGYN) at McMaster University (Hamilton, ON, Canada) prioritised the creation of an educational offering that:

1. Increases the competency-based learning opportunities afforded by simulation,
2. Maintains or improves resident learning through simulation,
3. Maintains or reduces the monetary and time costs associated with simulation,
4. Improves resident acceptance of simulation-based education.

To achieve these outcomes, a simulation-based curriculum that provides opportunities for appraising multiple trainees, at multiple levels, in learning activities that target professional activities associated with multiple competencies (Multi-TLC) across the CanMEDS 2015 spectrum [1] was developed, delivered, and evaluated. Specifically, the Multi-TLC required all residents to participate in simulated obstetrical emergencies in a variety of roles determined according to their level of training. The idea was that multiple trainees could be organised within the same scenario and address individual learning objectives simultaneously without competing for the same educational resources. We deemed this ‘learning concurrency’ and explicitly leveraged educational benefits associated with role play [2] and observational practice [3, 4] to achieve it, as well as aspects of deliberate practice [5], including the repetition of activities across scenarios, the setting of well-defined goals, and the delivery of immediate feedback. Furthermore, the deliberate practice model was also considered as we developed the curriculum so as to capture the essence of staged progression, which refers to the way in which the development of competence can be conceptualised as a sequential set of milestones that challenge trainees with greater degrees of complexity as they move through training [1]. This involved considering the medical expertise necessary for a learner to transition from first responder in an emergency to leader of an emergency team, while also understanding how development of other competencies are essential to this progression.

## Methods

### The innovation

**Fig. 1:**
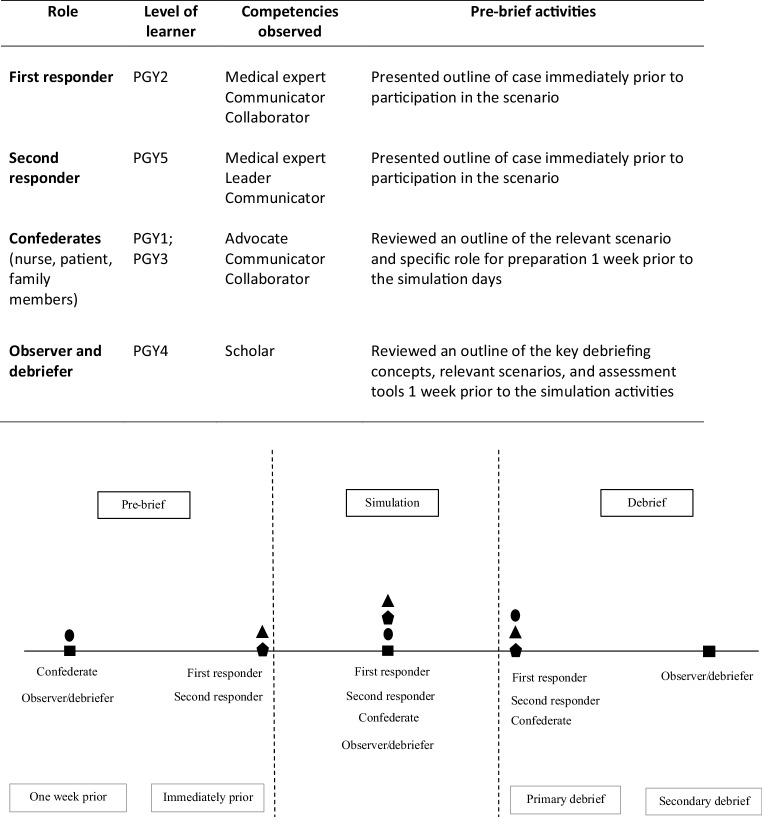
The first section presents a summary of the roles of learners, observed competencies, and pre-briefing activities for the simulations. The second section displays a schematic of the time-course of the pre-briefing, simulation, and debriefing activities for each group of learners

All residents participated in four simulated obstetrical emergency scenarios—postpartum haemorrhage, shoulder dystocia, eclampsia, and cord prolapse—in roles assigned according to their level of training. Each scenario took approximately 1.5 h, such that the entire Multi-TLC training curriculum was carried out over 8 h, spread evenly across 2 days (i.e., 4 h/day). The four scenarios ran simultaneously twice a day in order to accommodate the full cohort of trainees. Specifically:

1. A PGY2 resident participated in each scenario in the role of first responder, responsible for professional activities associated with recognising the emergency and initiating its management. Learning was facilitated through deliberate practice and role play. The medical expert and communicator competencies were observed.
2. Each scenario required a more experienced second responder—a PGY5 resident—to engage in professional activities associated with providing assistance to the first responder, assuming the role of team lead, and communicating effectively with other healthcare workers, patients, and family members. Learning was facilitated through deliberate practice and role play. The medical expert, communicator, and leader competencies were observed.
3. PGY1 and PGY3 residents served as confederates that acted as patients, nurses, and/or patient family members within the simulations, and participated in facilitated reflection on their experience during the debrief. These reflections formed the basis for conversations pertaining to professional activities involving communicator, collaborator, and health advocate competencies. Learning was facilitated by role play and observational practice.
4. A PGY4 resident participated alongside the instructor in professional activities associated with the scholar competency, which involved observing, providing feedback, and debriefing residents performing in medical expert roles. Learning was facilitated by observational practice (Fig. 1).

We acknowledged that instructors cannot provide feedback to multiple learners simultaneously, and organised the relevant observation and debriefing activities [6] in a sequential fashion. The instructor first observed residents involved in the management of the simulated emergency. These observations then served as the foundation for group debriefing on emergency management, during which confederate participants were invited to reflect on how the experience illuminates effective collaboration, communication, and advocacy. Assessor participants were invited to participate in the debriefing throughout and engaged with the instructor in a post-debriefing session concerned with delivering feedback (Fig. 1).

### The evaluation

#### Design

A comprehensive evaluation of the Multi-TLC curriculum was facilitated through application of the context-input-process-product model [7]. This report presents the outcomes of that evaluation in the form of quasi-experimental comparisons of the new and previous curricula with respect to the department’s four identified priorities for simulation-based education.

#### Participants

A total of 31 OBGYN residents (29 females, 2 males; 3 PGY1, 9 PGY2, 7 PGY3, 8 PGY4, 4 PGY5; average age = 30 ± 4.6 years) from McMaster University participated. All participants provided informed consent in accordance with the guidelines set forth by the Hamilton Integrated Research Ethics Board.

#### Data analyses

To demonstrate the increased breadth of competencies for which observations were afforded, we compared the number of CanMEDS 2015 competencies observed in the Multi-TLC to the department’s previous simulation curriculum.

To determine the impact of the Multi-TLC on resident demonstrations of competence within the simulation, we obtained a matched sample of 9 PGY2 and 4 PGY5 resident assessment scores from the postpartum haemorrhage emergency simulations that were completed in the most recent iteration of the previous curriculum and compared them against postpartum haemorrhage assessments for the Multi-TLC. The previous ratings were levied via modified Anaesthetist’s Non-Technical Skills (ANTS) tools [8], which included global performance assessments on 4‑point Likert scales. The ratings from the Multi-TLC simulation curriculum were levied for the first and second responder performances via tools with 9‑point ([9]; (Supplementary Material, Appendix 1)) and 7‑point ([10]; (Supplementary Material, Appendix 2)) global performance scores, respectively. To compare performances across the curricula, we converted the global assessments into proportions of the total possible score that were analysed in independent *t*-tests for the PGY2 and PGY5 learners.

The only financial cost associated with the previous simulation curriculum was the cost of the confederates (i.e., standardised patients) used for the scenarios. Thus, we identified the hourly rate for standardised patient confederates and multiplied that number by the length of time (1.5 h) it would take to run one resident per each scenario, and then multiplied that value by the number of simulations that would have been required to run 9 PGY2 and 4 PGY5 participants through four scenarios, and subtracted the resultant value from the total costs the Multi-TLC incurred.

In order to demonstrate the impact of the Multi-TLC on resident time, we calculated the total number of hours it would take to run each resident through each of the four simulations, given a per-scenario time requirement of 1.5 h. We then compared that value arithmetically against the number of hours per resident associated with the Multi-TLC approach.

In order to demonstrate the impact of the Multi-TLC on faculty time, we calculated the total number of faculty hours needed to run each of the four simulations (1.5 h long each) for each of our PGY2 and PGY5 residents, then subtracted that value from the total number of faculty hours associated with the Multi-TLC approach.

The evaluation also aimed to determine residents’ impressions of the curriculum. This involved semi-structured interviews with four focus groups defined by the participants’ roles within the simulations (first responder (*n* = 9); second responder (*n* = 4); confederate (*n* = 8); assessor (*n* = 8)). Transcribed interviews were appraised by two authors (V.M. and B.M.D.) using thematic analysis techniques adapted from constructivist methodological approaches [11].

## Results

The competencies observed in the previous simulation curriculum include medical expert, communicator, and collaborator. The competencies observed in the Multi-TLC include medical expert, communicator, collaborator, leader, advocate, and scholar (Tab. 1).

**Table 1:** Comparison between the previous curriculum and the and the Multi-TLC innovation

	Previous	Multi-TLC	Difference
Residents (*n*)	16	31	+15
Monetary costs (CAD)	$6240	0	−$6240
Faculty time (total)	78 h	32 h	−64 h
Resident time (per resident)	6 h	8 h	+2 h
Competencies assessed	3	6	+3

Comparison of converted postpartum haemorrhage scores from the previous curriculum to the Multi-TLC yielded no significant difference in the demonstration of medical expert competence for either the PGY2 residents (Previous = 0.813 (0.13); Multi-TLC = 0.835 (0.11); *t*(6) = 0.27, *p* = 0.80) or PGY5 residents (Previous = 0.938 (0.13); Multi-TLC = 0.833 (0.04); *t*(6) = 1.6, *p* = 0.16).

The cost associated with hiring actors ($40.00 (CAD) per hour × 2 confederates) to provide the same four scenarios (1.5 h per scenario × 4 scenarios) to 9 PGY2 and 4 PGY5 residents is $6240.00 (CAD). The Multi-TLC curriculum did not use actors and as such there were no direct departmental monetary costs (Tab. 1).

The total faculty instructor time required to observe, assess, and provide debriefing for the four scenarios (1.5 h × 4 scenarios) for each of 13 residents (9 PGY2; 4 PGY5) via the previous curriculum is 78 h. The total faculty time in the Multi-TLC for observation, assessment, and debriefing of 13 residents involved in simulated professional activities pertaining to the medical expert competency is 32 h (4 faculty × 4 h/ea. × 2 sessions).

The total time per resident spent in the scenarios and debriefing during in the previous curriculum was 6 h (1.5 h × 4 scenarios). The total time per resident spent in the scenarios and debriefing during the Multi-TLC was 8 h (4 h × 2 sessions).

The distillation of focus group data resulted in three thematic categories: ‘Meeting the CanMEDS competencies’; ‘Challenges to realism’; and ‘Giving and receiving feedback’.

The first theme ‘Meeting CanMEDS Competencies’ reflected the ways in which the Multi-TLC afforded the residents an opportunity to learn competencies outlined in the competency-based curriculum. In particular, trainees expressed that the experience benefited their ability to manage patients, and recognised that the simulation contributed to their development of ‘collaborator’ competencies. When asked about the value of having the paired PGY2 and PGY5 response teams, residents across all levels found this to be beneficial:

*I liked that we had split levels so senior and junior working together. [I]t is nice to learn from our seniors and see how we will develop in time.* (PGY2 #3)

*I think it was really a great experience for both of us, because they (PGY2) could ask questions, but we could also teach our juniors and maybe learn from things they did well.* (PGY5 #2)

Similarly, residents acting as confederates expressed that their participation in the simulations enhanced their understanding of the experiences of health professionals and, in turn, their future approach to inter-professional communication:

*It gives you a little insight too, into what it must be like to be a nurse with doctors yelling orders at you—this was a good learning experience for me.* (PGY2 #2)

The second theme, ‘Challenges to realism’, reflected both the positive and negative aspects of simulation-based learning, with primary attention focused on the challenges of maintaining realism in any simulation experience. In general, participants reflected that having a peer play a non-medical role compromised their perception of the realism of the simulation.

*I think it’s harder to make it as realistic when it’s your colleague.* (PGY5 #4)

The third theme, ‘Giving and receiving feedback’, reflected the varied learning that occurred through the different opportunities for providing and receiving feedback afforded by the experience. Indeed, one PGY2 participant described the specific value of having another resident play the confederate role in respect to the very specific feedback that they could provide:

*I think the benefit of them being your colleagues is that they are able to give constructive criticism because they do have that similar understanding.* (PGY2 #5)

In particular, the question of the appropriateness of delivering feedback ‘up-the-hierarchy’—from more junior to more senior trainee—was raised often. In doing so, the participant groups provided differing opinions. In particular, many of the PGY4 residents made comments reflecting their feeling that it was not necessarily right to provide feedback to residents who were their seniors.

*I think its fine to give feedback to the PGY2s but almost inappropriate to give feedback to the PGY5s in front of the juniors.* (PGY4 #3)

Interestingly, however, this sentiment was not shared by the PGY5 residents, who were generally in favour of the feedback:

*I feel open to receiving feedback from whomever … even the junior in my scenario.* (PGY5 #1)

## Discussion

In summary, the Multi-TLC increased the number of competencies trained and observed with indications that the increased breadth of objectives did not negatively impact resident performance within the simulations relative to the previous simulation curriculum. Of particular note, despite an increase in training and observation objectives, the change in structure of the simulation process resulted in significant reductions to departmental monetary costs and faculty time commitments. Furthermore, residents perceived the Multi-TLC curriculum as a positive learning experience that heightened their understanding of team-based emergent obstetrical care. The next step in this process is to evaluate the Multi-TLC as learners matriculate through each of the roles as they progress through training. This work will pay particular attention to the way learning at different levels is influenced by previous experiences within the simulation.

## Caption Electronic Supplementary Material

Example assessment tool for First Responders in the Multi-TLC simulation-based curriculum

Example assessment tool for Second Responders in the Multi-TLC simulation-based curriculum
