# Supplementary material for: A multiple-trainee, multiple-level, multiple-competency (multi-TLC) simulation-based approach to training obstetrical emergencies
Source: Perspect Med Educ. 2019 Oct 7;8(5):309–13. doi: 10.1007/s40037-019-00534-7 (PMC6820588; doi:10.1007/s40037-019-00534-7)
Supplement: Supplementary file 1 — Example assessment tool for First Responders in the Multi-TLC simulation-based curriculum [file 40037_2019_534_MOESM1_ESM.pdf]

OBSTETRICAL EMERGENCY SIMULATION – First Responders

Obstetrical Emergency: PPH
Date:
Resident:
Evaluator:

Critical Tasks

- Recognition
- Call for help
- CABs
  - Talk to and observe patient
  - Monitor vitals
  - Commence at least one large bore IV
  - Run crystalloid wide open
  - Obtain baseline BW (CBC, Cross match, coags, consider extended lytes and crea)
- Assess fundus
- Bimanual massage
- Foley catheter placement
- Utertonics
  - Oxytocin
  - Hemabate
  - Ergot
  - Misoprostol
- Bakri balloon
- Call for blood
- Communicate effectively with health care team

Please rate the following aspects of this applicant's performance on this station relative to all residents you are rating. Please place marks inside the boxes and not on the dividing lines.

1. Please score the resident's **communication skill** on this station:

|_______|_______|_______|_______|________|_______|_______|_______|______|
unacceptable marginal good excellent superior

2. Please score the strength of their **knowledge and skills** on this station:

|_______|_______|_______|_______|________|_______|_______|_______|______|
unacceptable marginal good excellent superior

3. Please score the applicant's **overall performance** on this station:

|_______|_______|_______|_______|________|_______|_______|_______|______|
unacceptable marginal good excellent superior

4. Please indicate areas where you feel the resident did well:
____
____

5. Please indicate areas where you feel the resident could improve:
____
____
