# Supplementary material for: A multiple-trainee, multiple-level, multiple-competency (multi-TLC) simulation-based approach to training obstetrical emergencies
Source: Perspect Med Educ. 2019 Oct 7;8(5):309–13. doi: 10.1007/s40037-019-00534-7 (PMC6820588; doi:10.1007/s40037-019-00534-7)
Supplement: Supplementary file 2 — Example assessment tool for Second Responders in the Multi-TLC simulation-based curriculum [file 40037_2019_534_MOESM2_ESM.pdf]

OBSTETRICAL EMERGENCY SIMULATION – Second Responders

Obstetrical Emergency: PPH
Date:
Resident:
Evaluator:

LEADERSHIP SKILLS
stays calm and in control during crisis
prompt and firm decision-making
Maintain global perspective ("Big picture")

SITUATIONAL AWARENESS
Avoids fixation error
Reassesses and re-evaluates situation constantly
anticipates likely events

INTERPROFESSIONAL COMMUNICATION SKILLS
Communicates clearly and concisely
listens to team input

PROBLEM SOLVING
Organized and efficient problem solving approach(ABC's)
Quick in implementation (Concurrent management)
Considers alternatives during crisis

RESOURCE UTILIZATION
Calls for help appropriately
utilizes resources at hand appropriately
Prioritizes tasks appropriately

OVERALL

OVERALL PERFORMANCE

1	2	3	4	5	6	7
Novice All CRM Skills require significant improvement		Advanced novice; many CRM skills require moderate improvement		Competent; most CRM skills require minor improvement		Clearly superior; few, if any skills require minor improvement

LEADERSHIP SKILLS

1	2	3	4	5	6	7
Loses calm and control for for entire or most of crisis; unable to make firm decisions without delay; cannot maintain global perspective		Loses calm and control frequently during crisis; delays in making firm decisions (or with cueing); rarely maintains global perspective		Stays calm and in control for most of crisis; makes firm decisions with little delay; usually maintains global perspective		Remains calm and in control; makes prompt and firm; always maintains global perspective

PROBLEM SOLVING SKILLS

1	2	3	4	5	6	7
Cannot implement ABC's without cues; always uses sequential management approach despite cues; fails to consider any alternatives		Incomplete or slow ABC assessment; mostly uses sequential management approach unless cued; gives little consideration to alternatives		Satisfactory ABC assessment without cues; mostly uses concurrent management approach with only minimal cueing; considers some alternatives		Thorough yet quick ABCs; concurrent management approach; considers most likely alternatives

SITUATIONAL AWARENESS SKILLS

1	2	3	4	5	6	7
Becomes fixated easily despite constantly repeated cues: fails to re-assess and re-evaluate situation despite repeated cues; fails to anticipate likely events		Avoids fixation error only with cueing; rarely reassesses and re-evaluates situation without cues; rarely anticipates likely events		Usually avoids fixation error with minimal cueing; reassesses & re-evaluates situation frequently with minimal cues; usually		Avoids any fixation error; reassesses and re-evaluates cues; constantly anticipates likely events

				anticipates likely events		

RESOURCE UTILIZATION SKILLS

1	2	3	4	5	6	7
Unable to use resources & staff; does not prioritize tasks or ask for help when required despite cues		Able to use resources with minimal effectiveness; only prioritize tasks or asks for help when required with cues		Able to use resources with moderate effectiveness; able to prioritize task and/or ask for help with minimal cues		Clearly able to utilize Resources effectively; sets clear task priorities; asks for help early with no cues

INTERPROFESSIONAL COMMUNICATION SKILLS

1	2	3	4	5	6	7
Does not communicate with team concisely at all times; does not acknowledge team feedback, never uses directed verbal/non-verbal communication		Communicates occasionally with team but, unclear and vague; occasionally listens to but rarely interacts with team; rarely uses directed verbal/non-verbal communication		Communicates with team clearly and concisely most of the time; listens to team feedback; usually uses verbal/nonverbal communication		Communicates clearly and encourages input and listens consistently uses directed communication

PATIENT COMMUNICATION SKILLS

1	2	3	4	5	6	7
Does not listen to patient/family; does not allow patient/family to express their feelings; does not show empathy		Occasionally actively listens to patients/family; rarely allows patient/family to express their feelings; rarely shows empathy		Usually listens to patient/family; allows patient/family to express their feelings most of the time; is empathetic most of the time		Always listens to patient/family; allows patient/family to express their feeling; always shows empathy
